# Comprehensive analysis of Alfin-like transcription factors associated with drought and salt stresses in wheat (*Triticum aestivum* L.)

**DOI:** 10.1186/s12864-024-10557-y

**Published:** 2024-07-17

**Authors:** Hao Liu, Wenyan Liu, Ziyi Wang, Na Li, Yongfeng Xie, Yanhong Zhao

**Affiliations:** 1 College of Agriculture, Ludong University, Yantai, 264000 China; 2 College of Agriculture, Henan University of Science and Technology, Luoyang, 471000 China; 3 College of Environment and Life Sciences, Weinan Normal University, Weinan, 714099 China

**Keywords:** Wheat, Genome-wide identification, Alfin-like transcription factor, Drought and salt stresses

## Abstract

**Background:**

Alfin-like proteins are a kind of plant-specific transcription factors, and play vital roles in plant growth, development and stress responses.

**Results:**

In this study, a total of 27 Alfin-like transcription factors were identified in wheat. *TaAL* genes were unevenly distributed on chromosome. Phylogenetic analysis showed *TaAL* genes were divided into AL-B and AL-C subfamilies, and TaALs with closer evolutionary relationships generally shared more similar exon-intron structures and conserved motifs. The *cis*-acting element analysis showed MBS, ABRE and CGTCA-motif were the most common in *TaAL* promoters. The interacting proteins and downstream target genes of *TaAL* genes were also investigated in wheat. The transcriptome data and real-time PCR results indicated *TaAL* genes were differentially expressed under drought and salt stresses, and *TaAL1-B* was significantly up-regulated in response to drought stress. In addition, association analysis revealed that *TaAL1-B-Hap-I* allelic variation had significantly higher survival rate compared to *TaAL1-B-Hap-II* under drought stress.

**Conclusions:**

These results will provide vital information to increase our understanding of the *Alfin-like* gene family in wheat, and help us in breeding better wheat varieties in the future.

**Supplementary Information:**

The online version contains supplementary material available at 10.1186/s12864-024-10557-y.

## Introduction

Wheat (*Triticum aestivum* L.) is one of the most important grain crops worldwide. As a sessile organism, wheat has to suffer from all kinds of adverse environmental factors, such as high salinity and drought, which seriously affect the yield and quality of wheat [1]. Therefore, plants have gradually developed intricate regulatory mechanisms over a long evolutionary period to avoid or defend against adverse environmental factors [2]. Transcription factors (TFs) are essential regulators in plant growth, development and stress responses through regulating the expression of the downstream target genes [3, 4].

The Alfin-like (AL) family proteins are a kind of plant-specific TFs [5]. AL proteins mainly contain two conserved domains, a conserved Alfin domain at the N-terminus consisting of 140 conserved amino acid residues and a PHD-finger domain with conserved Cys4-His-Cys3 (C4HC3) residues at the C-terminus [6, 7]. The PHD-finger are involved in epigenetics and chromatin-mediated transcriptional regulation [8, 9], which is responsible for the nuclear localization of the AL proteins [10, 11].

AL TFs have been identified to participate in various stress responses, such as salt, drought, and powdery mildew resistance [5–7, 12]. The first *AL* gene, *Alfin1*, was identified in alfalfa (*Medicago sativa* L.) [13], which could enhance plant root growth, and enhance salt tolerance via improving the expression of salt-induced *MsPRP2* gene [14, 15]. Overexpression of *Conringia planisiliqua CpAL2* gene conferred *Arabidopsis thaliana* seedlings with drought and salt resistance [16]. In *Arabidopsis*, *AtAL5* improved the salt, drought and freezing tolerance by binding to G-rich motifs (GTGGNG or GNGGTG) to regulate the expression of downstream stress-related genes [10]. Overexpression of *Atriplex hortensis AhAL1* gene enhanced drought and salt resistance in transgenic *Arabidopsis* plants [7]. Soybean (*Glycine max* (L.) Merr.) *AL* gene *GmPHD2* improved salt stress tolerance in transgenic *Arabidopsis* plants [17]. The heterologous expression of *MdAL4* gene improved the drought tolerance of transgenic *Arabidopsis* [6]. AL was also as a histone reader to bind to active histone mark H3K4me2/3 [18, 19]. AL2-PRC1 complexes promoted seed germination by active or repressive H3K4me3-to-H3K27me3 chromatin state switch, leading to the repression of seed developmental genes in *Arabidopsis* [20].

*Alfin-like* family genes have been identified in diverse plant species, such as *Arabidopsis* [10], rice (*Oryza sativa* L.) [5], apple (*Malus domestica*) [6], and Chinese cabbage (*Brassica rapa*) [21]. However, a comprehensive analysis of *Alfin-like* gene family in wheat has not been investigated. In present study, a genome-wide identification of *Alfin-like* genes in wheat was performed, and their physicochemical properties, gene structure, conserved motif, evolutionary relationship, chromosome distribution, gene duplication and regulatory network were also analyzed. Furthermore, the tissue-specific and expression patterns under drought and salt stresses of *Alfin-like* gene were determined. In addition, we used wheat resequencing data from Wheat Union database [22] and drought-related trait data acquired from published study [23] to perform association analysis with *TaAL-B* gene. These results will provide a valuable foundation for further functional study of *Alfin-like* genes in wheat.

## Results

### Identification and phylogenetic analysis of *Alfin-like* genes

**Fig. 1:**
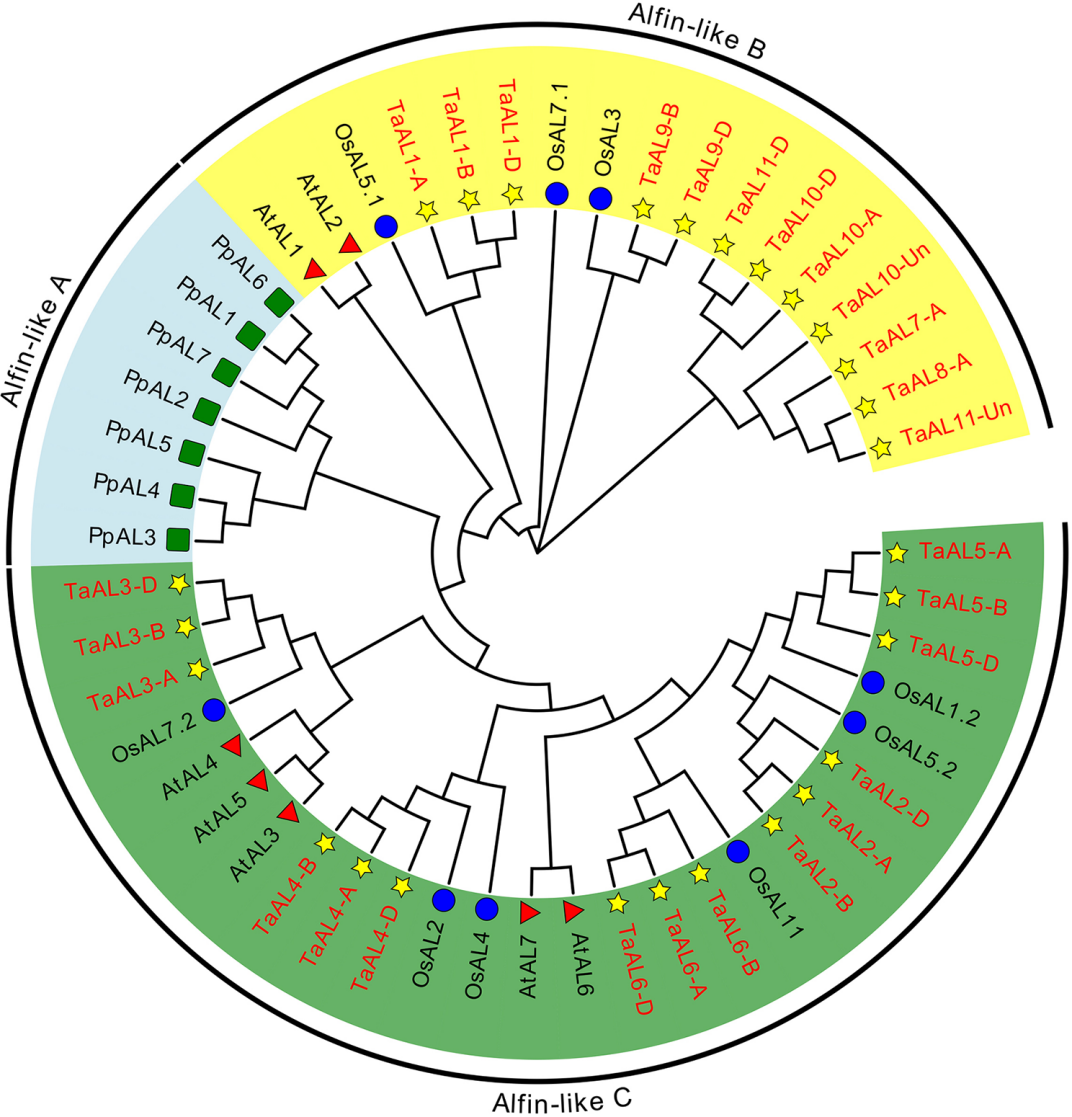
The neighbor-joining (NJ) phylogenetic tree of AL proteins. The phylogenetic tree was constructed with AL protein sequences from *Arabidopsis* (At), *Oryza sativa* (Os), *Physcomitrella patens* (Pp), and *Triticum aestivum* (Ta) with bootstrap values of 1000 replicates. The AL proteins are classified into three subfamilies: Alfin-like A (AL-A), Alfin-like A (AL-B), and Alfin-like A (AL-C). AL protein members of different subfamilies are distinguished by different colors

Through a Hidden Markov Model (HMM) search, a total of 27 members of *Alfin-like* gene family were identified in wheat, and renamed them according to their collinearity analysis and subgenome distribution (Table S1 and Fig. 1). The phylogenetic tree was constructed with AL protein sequences from moss (*Physcomitrella patens*, 7 PpALs), dicot (*Arabidopsis*, 7 AtALs), and monocot (*Oryza sativa*, 10 OsAls; *Triticum aestivum*, 27 TaALs) (Fig. 1). Based on the phylogenetic relationship of AL proteins, they were classified into three subfamilies: AL-A, AL-B, and AL-C (Fig. 1). AL-B and AL-C subfamilies included twelve and fifteen TaAL protein members, respectively, whereas no TaAL protein member belonged to AL-A subfamily (Fig. 1 and Table S1). AL-A members only existed in ancient plant species, e.g., *P. patens*. AL-B and AL-C were present in higher plants such as monocotyledonous and dicotyledonous plants [5]. Interestingly, AL-B members in dicot (AtALs) and monocot (OsALs and TaALs) were clustered in separate branches (Fig. 1), suggesting that AL-B proteins occurred after differentiation of the monocot and dicot. In contrast, AL-C members might occur before differentiation of the monocot and dicot. These results suggested that AL proteins evolved with the evolution of plants from ancient plant to higher plant species, and AL-B and AL-C proteins evolved independently in higher plant species.

### Characterization, gene structure and conserved motif analysis of *TaAL* genes

The length of the identified 27 TaAL proteins ranged from 219 to 304 amino acid residues, and the molecular weights ranged from 24.95 to 34.6 kDa. The *p*I values varied from 4.55 (TaAL10-A) to 6.31 (TaAL9-B), with the calculated grand average of hydrophilic index (GRAVY) ranging from − 0.864 (TaAL3-A) to -0.489 (TaAL4-B), suggesting that these 27 *TaAL* genes encoded highly hydrophilic proteins. The subcellular localization prediction suggested all TaALs were located in the nucleus (Table S1).

**Fig. 2:**
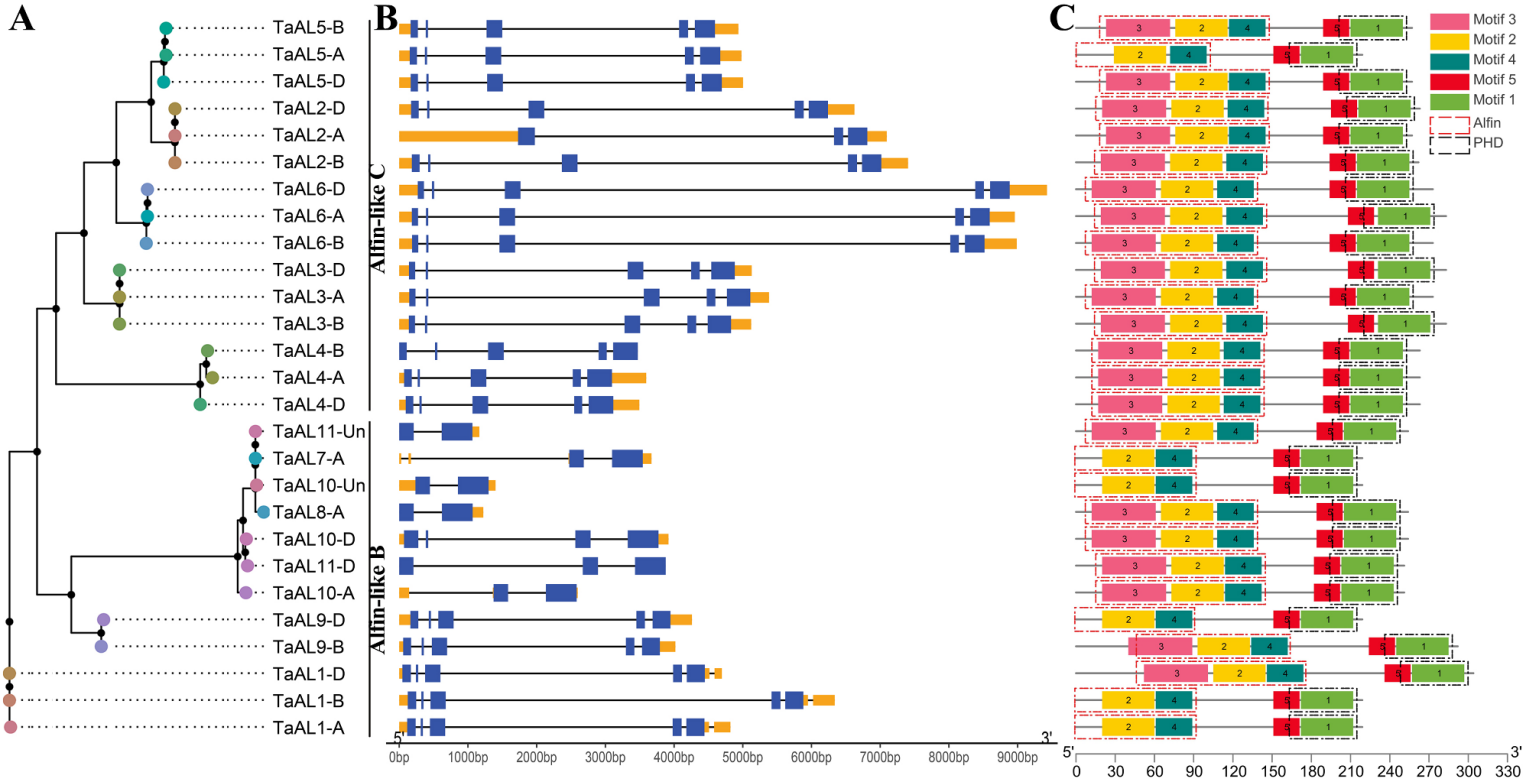
Phylogenetic classification, exon–intron structures and conserved domains of *TaAL* genes. (**A**) Phylogenetic analysis of *TaAL* gene family. (**B**) The introns, exons and UTR are represented by black lines, blue boxes and yellow boxes, respectively. (**C**) Each motif is represented using colored boxes

To investigate the structural characteristic of *TaAL* genes, the exon–intron structures and conserved motifs were analyzed (Fig. 2). The exon number of *AL-B* genes varied from 2 to 5. *AL-C* subfamily genes included five exons and four introns, except for *TaAL2-A* contained three exons and two introns (Fig. 2B). Meanwhile, five conserved motifs were identified among TaAL proteins by using the MEME program (Fig. 2C). All TaALs contained motif 1, 2, 4, and 5. Motif 2, 3 and 4 formed Alfin domain, and motif 1 and 5 made up PHD domain (Fig. 2C and Fig. S1). These results also verified the reliability of the identified TaAL proteins. TaALs with closer evolutionary relationships generally shared more similar exon-intron structures and conserved motifs (Fig. 2).

### Chromosomal location, collinearity and Ka/Ks analysis of *TaAL* genes

**Fig. 3:**
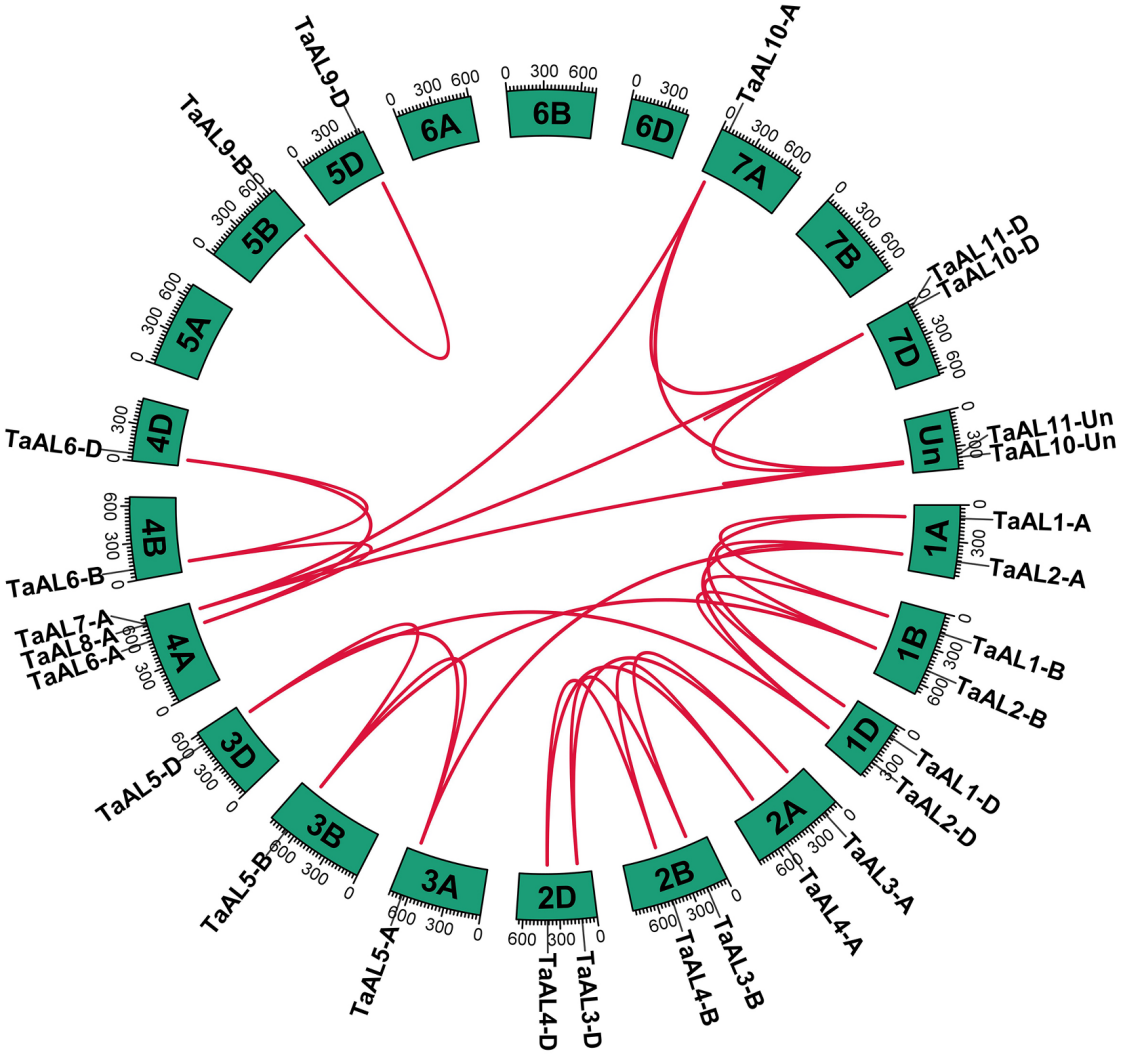
Chromosomal localizations and syntenic relationships among *TaAL* genes in wheat. Red lines in the highlight indicate the syntenic TaAL gene pairs

The chromosomal location and collinearity of *TaAL* genes were analyzed according to their genomic information (Fig. 3 and Fig. S2). *TaAL* genes were unevenly distributed on chromosome 1 A, 1B, 1D, 2 A, 2B, 2D, 3 A, 3B, 3D, 4 A, 4B, 4D, 5B, 5D, 7 A, 7D, and Un (Fig. S2). To investigate the expansion and evolution of *Alfin-like* family genes in wheat, a collinearity analysis of *TaAl*s was performed using TGT (Triticeae-Gene Tribe) website (Fig. 3). A total of thirty-five paralogous gene pairs of *TaAL* genes were identified in wheat genome. Expect *TaAL7-A*, *TaAL8-A*, *TaAL10-D* and *TaAL11-D* were tandem duplicate and block duplicate, the other *TaAL* genes all undergone block duplicate events (Table S2). Ka/Ks (the nonsynonymous and synonymous substitution ratio) values of four paralogous gene pairs (*TaAL8-A*/*TaAL10-D*, *TaAL8-A*/*TaAL11-Un*, *TaAL10-D*/*TaAL11-D*, *TaAL10-A*/*TaAL11-D*) were more than 1, suggesting these genes have undergone positive selection after duplication events (Table S2). And the Ka/Ks ratio of the other thirty-one paralogous gene pairs were less than 1, suggesting these thirty-one paralogous gene pairs undergone purifying selection to maintain the function of *TaAL* gene family (Table S2).

### Analysis of *cis*-acting elements of *TaAL* promoters

**Fig. 4:**
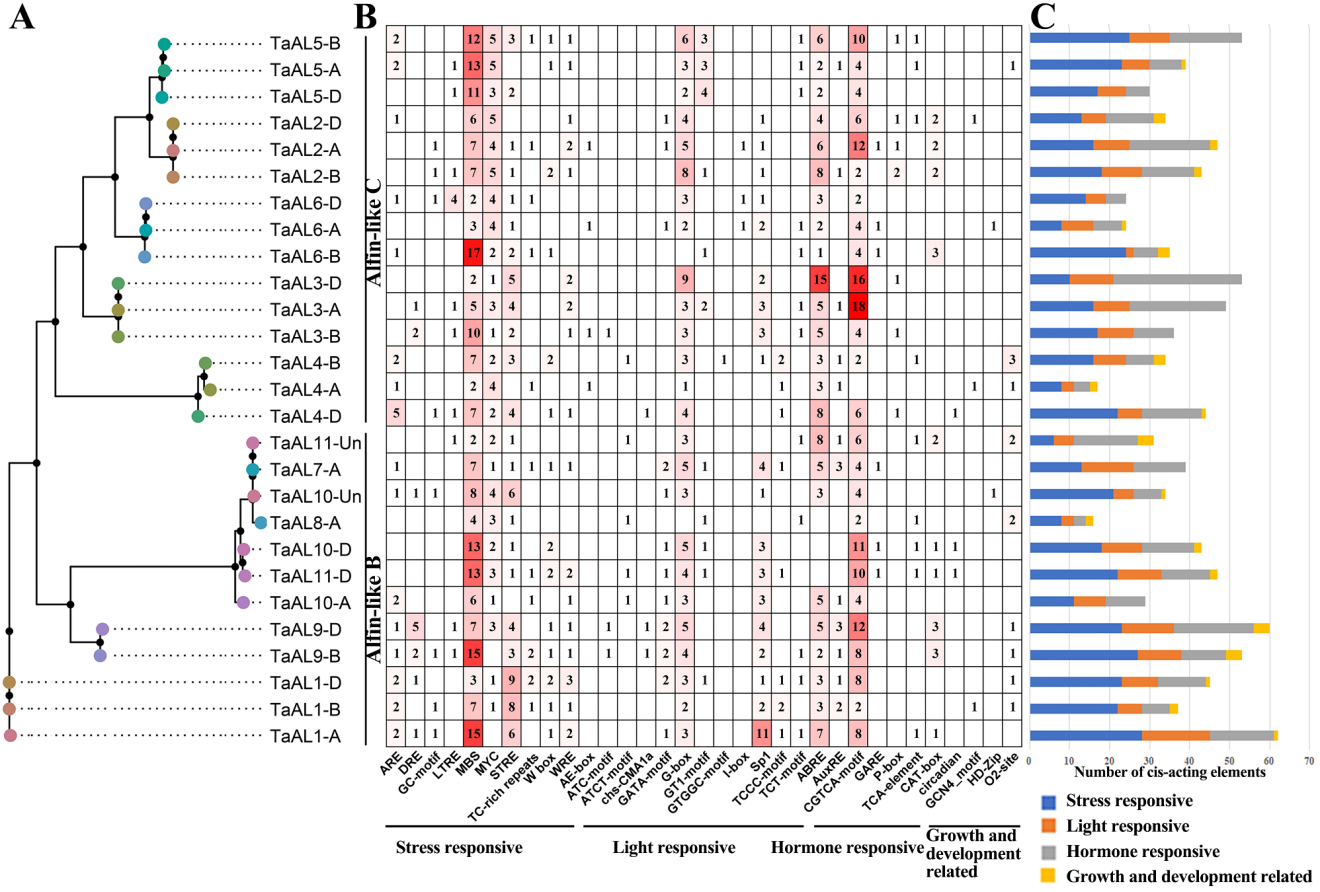
Analysis of *cis* -acting elements in *TaAL*promoters. (**A**) Phylogenetic analysis of *TaAL* gene family. (**B**) Cis-acting elements in the promoters of *TaAL* genes. The different colors and numbers of grids indicated the numbers of different promoter elements. (**C**) The histograms of different colors represented the sum of the cis-acting elements in each category

The variable *cis*-acting elements in gene promoters can bind to various transcription factors to regulate gene expression, thus revealing the differences biological function of genes. To study the function of *TaAL* genes, 1500 bp promoter sequences of *TaAL*s were analyzed through PlantCARE database to identify the *cis*-acting elements (Fig. 4). A total of 33 *cis*-acting elements were identified and divided into four categories, including ten stress responsive-elements, six hormone responsive-elements, twelve light responsive-elements, and five growth and development related-elements. The *TaAL* promoters mainly contained stress responsive- and hormone responsive-elements, especially MBS (MYB binding site), ABRE (ABA-responsive element) and CGTCA-motif (MeJA-responsive element) were the most common in *TaAL* promoters (Fig. 4). These results suggested that *TaAL* genes might play crucial roles in response to various stresses in wheat.

### Expression profiles of *TaAL*s

**Fig. 5:**
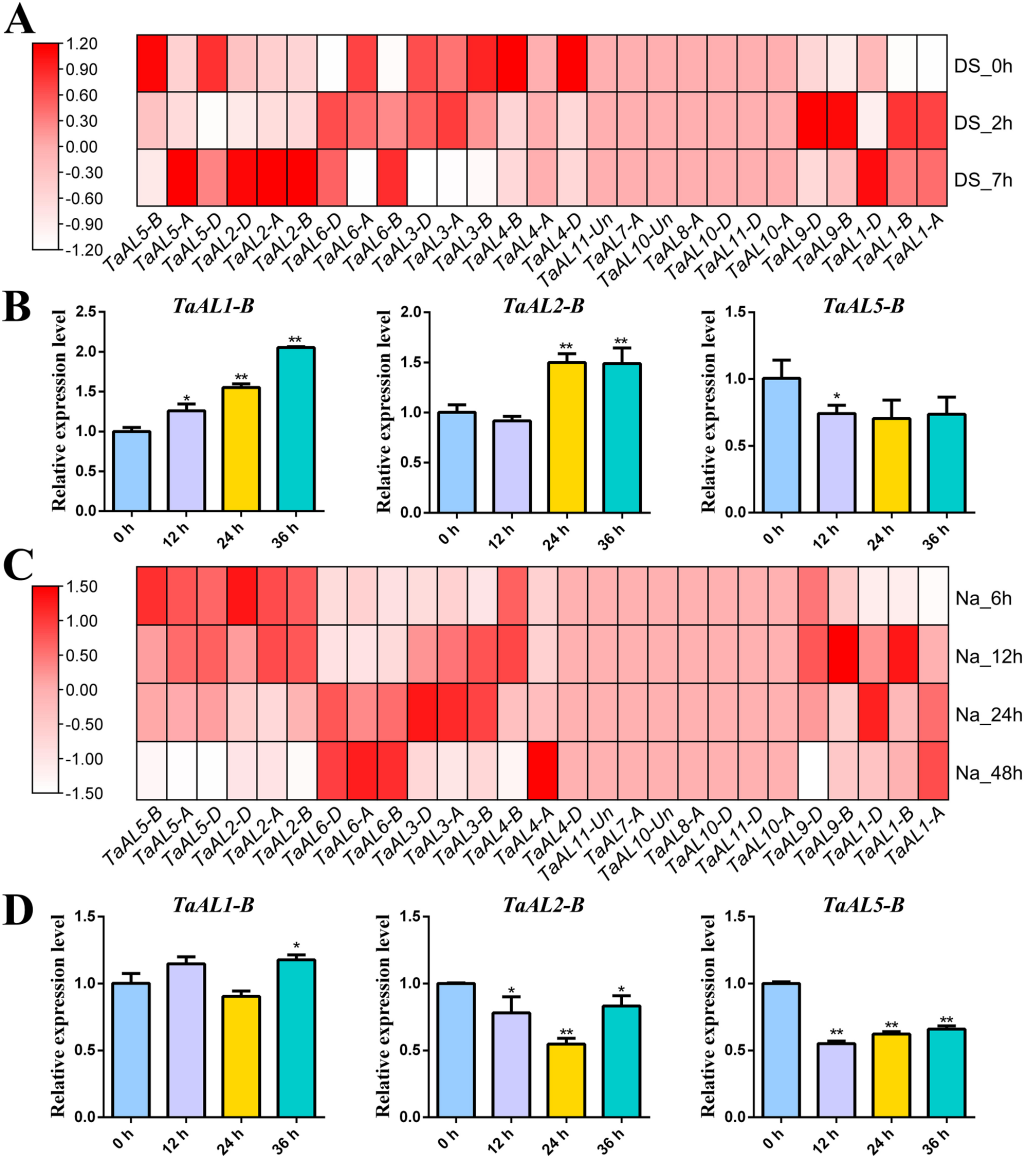
Expression patterns of *TaAL* genes in response to drought and salt stresses. (**A**) RNA-seq analysis of the expression profiles of *TaAL* genes in response to drought stress for 0 h, 2 h, and 7 h, respectively. Fragments per kilobase of exon per million mapped fragments (FPKM) values were used to measure the expression levels of genes. (**B**) Real-time PCR analysis of the expression profiles of *TaAL* genes in wheat seedling leaves at 0 h, 12 h, 24 h, and 36 h after PEG stress treatment.(**C**) RNA-seq analysis of the expression profiles of *TaAL* genes in response to salt stress for 6 h, 12 h, 24 h, and 36 h, respectively. Fragments per kilobase of exon per million mapped fragments (FPKM) values were used to measure the expression levels of genes. (**D**) Real-time PCR analysis of the expression profiles of *TaAL* genes in wheat seedling leaves at 0 h, 12 h, 24 h, and 36 h after salt stress treatment. The values show the mean ± SD. Significant statistical analysis was carried out by Student’s t -test (*p < 0.05; **p < 0.01)

The expression profiles of the *TaAL* genes in different tissues and under drought and salt stresses during the wheat seedling stage were determined by using transcriptome data and real-time PCR (Fig. 5 and Fig. S3). The results showed *TaAL1-B*, *TaAL2-B* and *TaAL5-B* were expressed at a higher level in shoot tissues and at a lower level in root tissues (Fig. S3). The transcriptome data revealed that the expression levels of *TaAL1-A*/*B*/*D*, *TaAL2-A*/*B*/*D*, *TaAL5-A*, *TaAL6-B*/*D* and *TaAL9-B*/*D* were significantly up-regulated under drought stress, and *TaAL3-A*/*B*/*D*, *TaAL4- B*/*D*, *TaAL5-B*/*D*, *TaAL6-A* were down-regulated (Fig. 5A and Table S3). Consistently, real-time PCR results showed that the expression levels of *TaAL1-B* and *TaAL2-B* were up-regulated and reached the highest expression level at 36 h and 24 h after PEG-induced drought stress with approximately 2.05- and 1.50-fold compared with the control, respectively. *TaAL5-B* was down-regulated under PEG stress (Fig. 5B). After salt stress treatment, the expression levels of *TaAL1-A*/*B*/*D*, *TaAL3-A*/*B*/*D*, *TaAL6-A*/*B*/*D*, *TaAL4-A* and *TaAL9-B* were obviously up-regulated, and *TaAL2-A*/*B*/*D*, *TaAL4-B*, *TaAL5-A*/*B*/*D*, and *TaAL9-D* were down-regulated (Fig. 5C and Table S3). The real-time PCR results also showed that *TaAL2-B* and *TaAL5-B* were down-regulated under salt stress (Fig. 5D). These results suggested that *TaAL* genes had different expression levels under drought and salt stresses and might be involved in the regulatory pathways of drought and salt stresses.

### The interacting proteins and downstream target genes analysis of *TaAL* genes

**Fig. 6:**
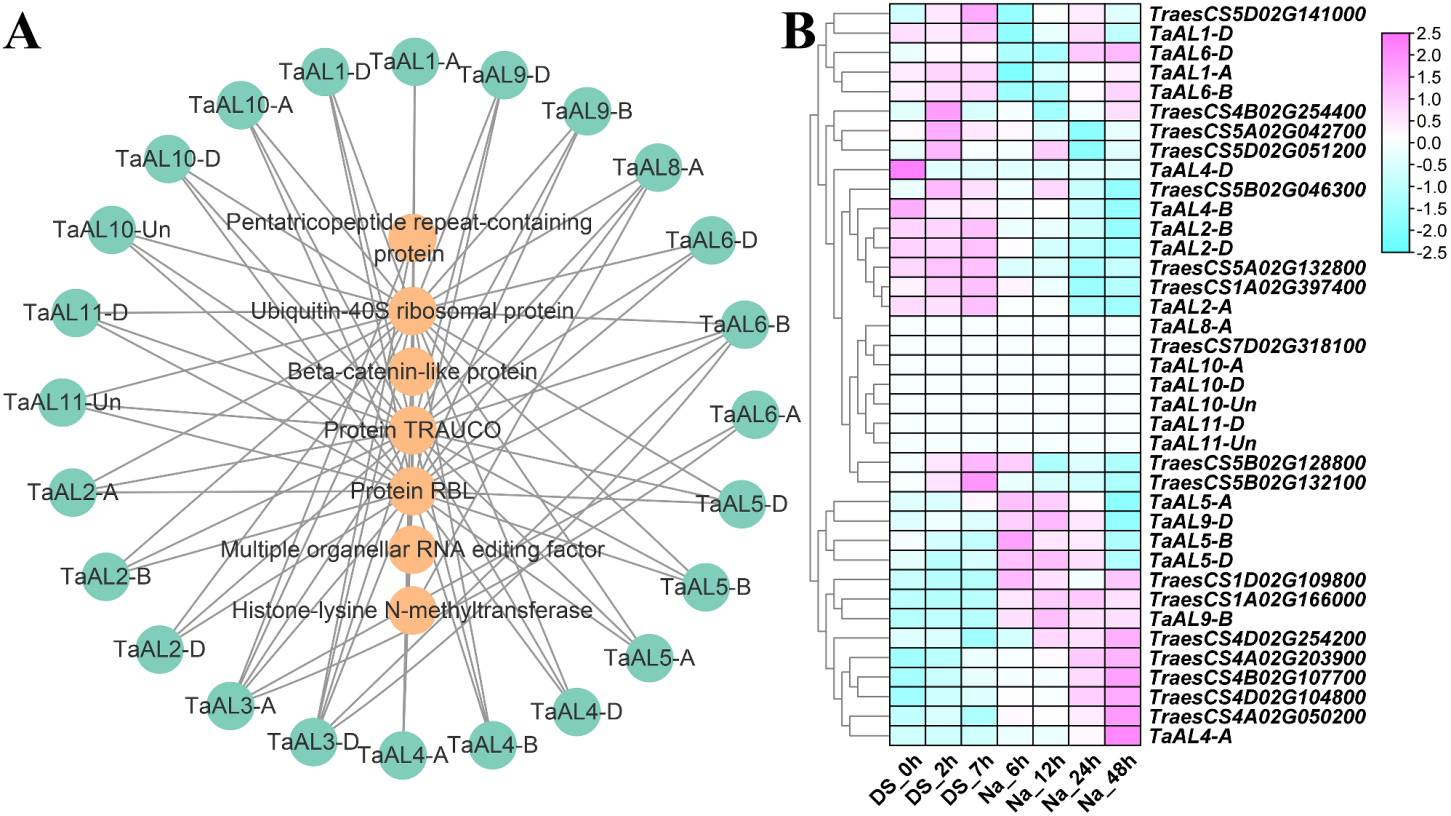
Protein–protein interaction (**A**) and expression profiles analysis of *TaAL*. Fragments per kilobase of exon per million mapped fragments (FPKM) values were used to measure the expression levels of genes

**Fig. 7:**
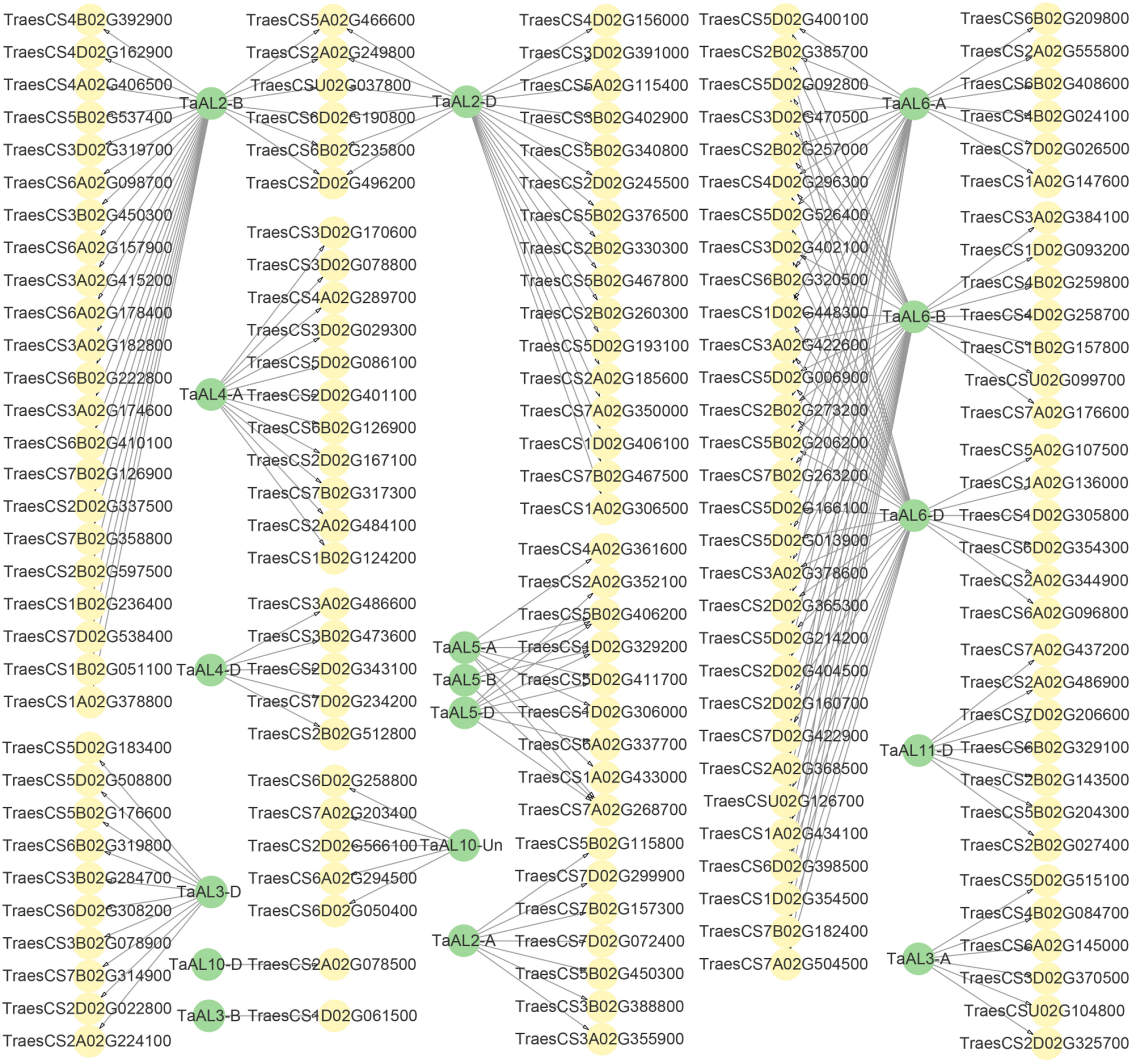
The downstream target gene of TaAL proteins

To determine the regulatory mechanism of *TaAL* genes in response to drought and salt stresses, the interacting proteins and downstream target genes of *TaAL*s were analyzed (Figs. 6 and 7). A total of 25 interacting proteins of TaAL were identified by using the STRING [24] and plant.MAP [25] database, including Alfin-like transcription factor, beta-catenin-like protein, histone-lysine N-methyltransferase, multiple organellar RNA editing factor, pentatricopeptide repeat-containing protein, protein RBL, protein TRAUCO, and ubiquitin-40 S ribosomal protein (Fig. 6A and Table S4). Cluster analysis of gene expression showed *TaAL1-A*/*D* exhibited the most similar expression patterns with interacting protein TRAUCO (*TraesCS5D02G141000*) under drought and salt stresses. *TaAL2-A*/*B*/*D* had the most similar expression patterns with TRAUCO (*TraesCS5A02G132800*) and ubiquitin-40 S ribosomal protein (*TraesCS1A02G397400*). *TaAL5-A*/*B*/*D* showed the most similar expression patterns with ubiquitin-40 S ribosomal protein (*TraesCS1D02G109800*, and *TraesCS1A02G166000*) (Fig. 6B). These interacting proteins that have similar expression patterns with *TaAL*s might perform important roles through interacting with TaAL proteins under drought and salt stresses.

As transcription factors, TaALs could regulate downstream target genes in response to drought and salt stresses. Therefore, the downstream target genes of TaALs were obtained through identifying the promoters of co-expression genes including G-rich motifs (Table S5 and S6). The results showed the downstream target genes of TaAL2-A (7), TaAL2-B (28), TaAL2-D (22), TaAL3-A (6), TaAL3-B (1), TaAL3-D (10), TaAL4-A (11), TaAL4-D (5), TaAL5-A (7), TaAL5-B (5), TaAL5-D (7), TaAL6-A (27), TaAL6-B (34), TaAL6-D (31), TaAL10-D (1), TaAL10-Un (5) and TaAL11-D (7) were identified (Fig. 7 and Table S5). The expression patterns of *TaAL*s and their downstream target genes under drought and salt stresses were also analyzed (Fig. S4). Cluster analysis of gene expression showed *TaAL2-A*, *TaAL2-B* and *TaAL2-D* had the most similar expression patterns with chromatin remodeling protein (*TraesCS5B02G450300*), actin-related protein (*TraesCS2B02G597500*) and E3 ubiquitin-protein ligase (*TraesCS7A02G350000*), respectively (Fig. 7 and Fig. S4). Additionally, the most similar expression patterns were observed between TaAL5-A with upstream activation factor subunit (*TraesCS5D02G411700*), TaAL5-B with bZIP transcription factor (*TraesCS7A02G268700*) and upstream activation factor subunit (*TraesCS5B02G406200*), TaAL5-D with bZIP transcription factor (*TraesCS2A02G352100*). These genes that have similar expression patterns with *TaAL*s might perform important roles through regulating the expression by TaAL under drought and salt stresses.

### Association analysis of *TaAL1-B* gene haplotypes with drought-related traits in wheat

**Fig. 8:**
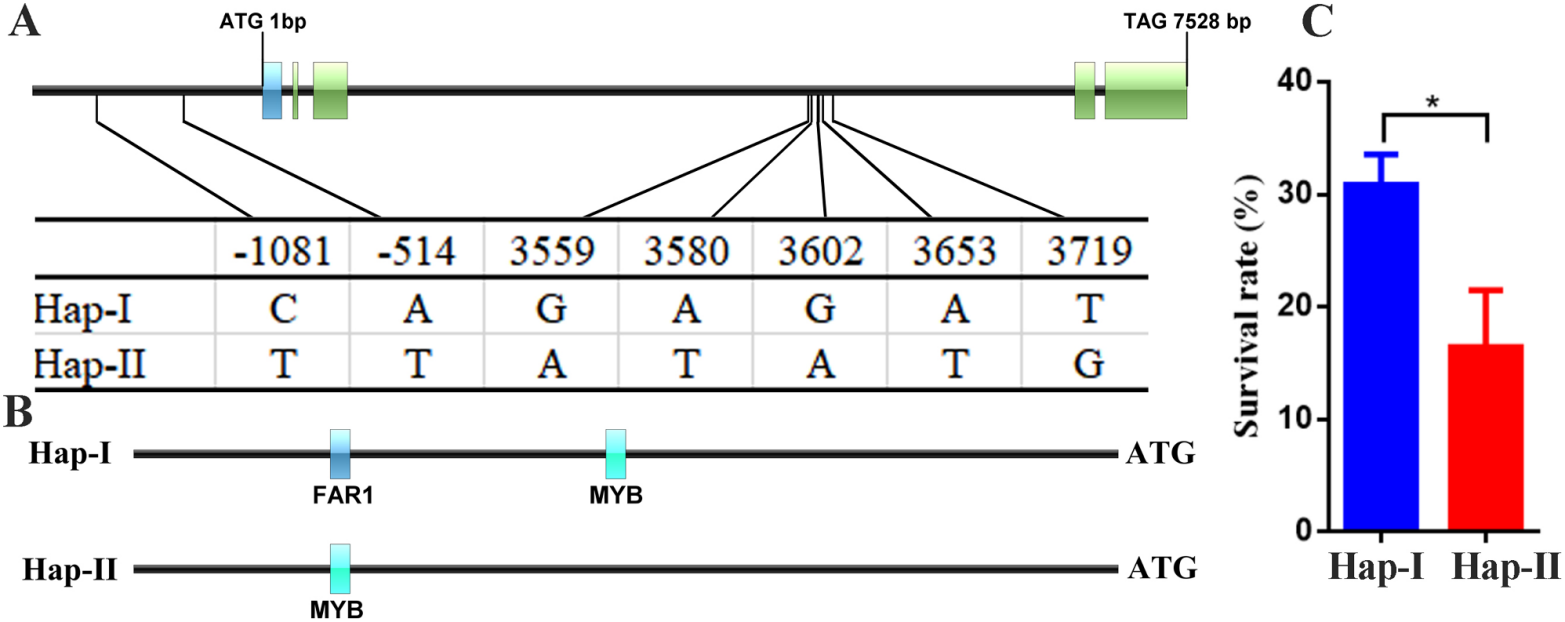
Association analysis of *TaAL1-B* gene haplotypes with drought-related traits in wheat. (**A**) *TaAL1-B* gene structure and SNP sites of the two haplotypes. (**B**) Distribution of transcription factor binding site in two haplotype promoters of *TaAL1-B* gene. (**C**) Association analysis of two haplotypes of *TaAL1-B* with survival rate under drought stress. The values show the mean ± SD. Significant statistical analysis was carried out by Student’s t -test (*p < 0.05; **p < 0.01)

According to transcriptome data and real-time PCR results, *TaAL1-B* gene was highly expressed under drought stress. Therefore, we detected the variation sites in 1500 bp promoter and CDS (coding sequence) region of *TaAL1-B* gene by using 681 wheat resequencing data in Wheat Union database (Table S7). The results showed that 7 SNP (single-nucleotide polymorphism) variants were detected, i.e., C/T (-1081 bp) and A/T (-514 bp) were located in promoter region, G/A (3559 bp), A/T (3580 bp), G/A (3602 bp), A/T (3653 bp), and T/G (3719 bp) were located in intron region (Fig. 8A). Based on these SNP variants, two haplotypes were identified and named *TaAL1-B-Hap-I* and *TaAL1-B-Hap-II* (Fig. 8A and Table S8). Due to SNP variants, FAR1 (far-red impaired response 1) TF could bind to *TaAL1-B-Hap-I* promoter at -1081 bp, however, MYB TF bound to *TaAL1-B-Hap-II* promoter at -1081 bp. Otherwise, MYB TF could bind to *TaAL1-B-Hap-I* promoter at -514 bp, but no TF was detected to bind to *TaAL1-B-Hap-II* promoter at -514 bp (Fig. 8B). To analysis the effect of *TaAL1-B* allele variation on survival rate under drought stress in wheat, we used published data from 39 of these 681 wheat varieties to conduct the associations analysis of *TaAL1-B* genes with survival rate under drought stress (Table S9). The results showed *TaAL1-B-Hap-I* allelic variation had significantly higher survival rate compared to *TaAL1-B-Hap-II* under drought stress (Fig. 8B and Table S9). Therefore, the transcription factor binding site in the promoter of *TaAL1-B* gene with *Hap-I* might be involved in regulating wheat drought tolerance.

## Discussion

Alfin-like transcription factors play crucial roles in plant growth, development and stress responses [26]. In this study, 27 *TaAL* genes were identified and comprehensively analyzed the roles of Alfin-like transcription factors in wheat (Fig. 1 and Table S1). The phylogenetic analysis showed that *AL* genes were classified into *AL-A*, *AL-B* and *AL-C* subfamilies, and *TaAL* genes belonged to AL-B and AL-C members (Fig. 1). *AL-A* subfamily genes only existed in ancient plant species, and AL-B members occurred after differentiation of the monocot and dicot, which was consistent with previous study [5]. Most *TaAL* genes undergone block duplicate events, which played vital function for expansion of *TaAL* gene family (Table S2). This was consistent with results of the *AL* gene family in *Malus domestica*, *Arabidopsis lyrata*, *Arabidopsis thaliana*, and *Thellungiella halophila* [6, 27].

Previous studies showed that the *AL* genes participated in regulating plant growth, development and stress responses [5, 6, 16, 28]. In order to investigate the function of *TaAL*s, we analyzed the *cis*-acting elements in the promoter of *TaAL*s, the stress responsive-elements, hormone responsive-elements, light responsive-elements, and growth and development related-elements were identified (Fig. 4). In light responsive-element category, G-box had the largest number, suggesting the interaction of G-box with TFs facilitated the involvement of *TaAL*s in light signaling. Similarly, *TaAL*s might participate in ABA and MeJA signaling through the interaction of ABRE and CGTCA-motif with TFs. *AhAL1* was up-regulated after ABA treatment and could enhance the ABA-mediated stomatal closure [7]. In addition, *AL* genes were involved in root hair elongation and seed germination [7, 26], which might be regulated by hormone responsive- and growth and development related-elements. Most *TaAL* promoter included MBS, ABRE and CGTCA-motif (Fig. 4). MBS and ABRE elements were related to drought stress response [29, 30]. Thus, the expression level of *TaAL* genes were studied under drought and salt stresses. The transcriptome data revealed up-regulation of *TaAL1-A/B/D*, *TaAL6-B/D* and *TaAL9-B* under drought and salt stresses, suggesting their potential crosstalk between drought and salt stresses. The real-time PCR results showed *TaAL1-B* was significantly up-regulated in response to drought stress (Fig. 5 and Table S3).

The interacting protein and cluster analysis showed that *TaAL*s were most likely to interact with TRAUCO and ubiquitin-40 S ribosomal protein under drought and salt stresses (Fig. 6). TRAUCO protein was a core component of histone methyltransferase complex [31], and AL was a histone reader to bind to histone mark H3K4me2/3 [18, 19]. In *Arabidopsis*, AL2-PRC1 complexes promoted seed germination through H3K4me3-to-H3K27me3 chromatin state switch in repression of seed developmental genes [20]. Therefore, the interaction of TaAL with TRAUCO protein might regulate transcription initiation of target genes by changing chromatin state to respond drought and salt stresses. TaAL might participate in protein degradation by interacting with ubiquitin-40 S ribosomal protein. The downstream target genes and cluster analysis showed that TaAL might regulate the expression of chromatin remodeling protein, actin-related protein, E3 ubiquitin-protein ligase, upstream activation factor subunit, and bZIP transcription factor gene to respond drought or salt stresses (Fig. 7 and Fig. S4). These target genes played important roles in response to drought or salt stresses [32–35].

Association analysis of haplotypes with drought-related traits showed that *Hap-I* allelic variation of *TaAL1-B* had significantly higher survival rate compared to *Hap-II* under drought stress, probably due to the difference of transcription factor binding site in the promoter of *TaAL1-B* haplotypes lead to the difference of wheat drought tolerance (Fig. 8B and Table S9). FAR1 and MYB bound to *TaAL1-B-Hap-I* and *Hap-II* promoters, respectively (Fig. 8). In *Arabidopsis*, *FAR1* could be up-regulated by ABA and drought stresses [36]. The *far1* mutants had wider stomata, lose water faster, and were more sensitive to drought than the wild type plants [36]. Overexpression of *HvFRF9* significantly enhanced the drought tolerance in *Arabidopsis* by increasing the absorption and transportation of water and the activity of antioxidant enzymes [37]. *FAR1* negatively controlled ROS levels by directly or indirectly regulating genes involved in ROS homeostasis [38]. Therefore, the up-regulation of *FAR1* might regulate the expression of *TaAL1-B-Hap-I* to enhance drought resistance by regulating ROS homeostasis. Furthermore, *FAR1* was not only involved in light signaling, but also the positive regulator of ABA signaling, which was a hub for the integration of light and ABA signaling pathway [36, 38]. *FAR1* might also enhance drought resistance via ABA signaling pathway [38]. Similarly, the SNPs were identified in the promoter region of *OsAL7.1*, which were significantly associated with grain yield, drought coefficient and seed width [5]. These results lay a foundation for further functional studies of Alfin-like transcription factors under drought and salt stresses.

## Materials and methods

### Genome-wide identification of *Alfin-like* family genes

The candidate Alfin-like protein sequences were obtained by using the PFAM ID of Alfin domain (PF12165) [39] to search against wheat protein database in WheatOmics 1.0 website [40]. Then, the InterPro [39] and SMART (Simple Modular Architecture Research Tool) [41] database were used to confirm the candidate Alfin-like proteins. The physiological and biochemical parameters of the Alfin-like proteins were analyzed by WheatOmics 1.0 [40]. The subcellular localization of the Alfin-like proteins were predicted using Plant-mPLoc [42].

### Multiple sequence alignment and phylogenetic tree construction

Multiple sequence alignment of Alfin-like amino acid sequences was performed with ClustalW using the default options in MEGA 11 [43] and visualized by ESPript 3.0 [44]. The phylogenetic tree was constructed by using the neighbor-joining (NJ) method with 1000 bootstrap replicates in MEGA 11 software [43] and visualized by Evolview service [45].

### Gene structures, conserved motifs and domains analyses

The exon-intron structures of *Alfin-like* genes were analyzed based on TGT (Triticeae-Gene Tribe) [46]. The conserved motifs and domains of Alfin-like family proteins were annotated using the MEME program [47] and SMART database [41], and visualized by the TBtools [48].

### Chromosome localization, synteny and *Ka*/*Ks* analyses

The chromosome localization and paralogous gene pairs of *Alfin-like* genes were identified by using TGT (Triticeae-Gene Tribe) [46]. The gene duplication events were determined by PLAZA database [49]. TBtools was used to calculate the *Ka* (non-synonymous rate), *Ks* (synonymous rate), and *Ka*/*Ks* (the nonsynonymous and synonymous substitution ratio) values of the paralogous gene pair with the Nei-Gojobori (NG) method [48].

### Analysis of *cis*-acting elements of *TaAL* promoters

The 1500 bp promoter sequences of *TaAL* genes were obtained from the wheat genome database [40]. PlantCARE database (https://bioinformatics.psb.ugent.be/webtools/plantcare/html/) was used to identify and count the *cis*-acting elements on the *TaAL* genes promoter.

### The interacting proteins and downstream target genes prediction

Protein–protein interaction (PPI) was analyzed using the STRING [24] and plant.MAP [25] database. To predict the downstream target genes of *TaAL*s, WheatCENet database [50] was used to obtain the co-expression genes associated with *TaAL*s (PCC > = 0.9), and the co-expression genes that including G-rich motifs (GNGGTG/GTGGNG) [15] on 1.5 kb promoter sequences were potential downstream target genes of *TaAL*s.

### Transcriptome data analysis

The transcriptome data of NCBI SRA (SRX2948720, SRX2948721, SRX2948729, SRX2948726, SRX2948731, SRX2948728, SRX2948715, SRX2948717, and SRX2948727) were used to analyze wheat gene expression profiles under drought stress. NCBI SRA data (SRX1162592, SRX1162594, SRX1162596, and SRX1162598) were obtained to analyze wheat gene expression profiles under salt stress.

### RNA extraction and real-time PCR

Real-time PCR was performed to detect the expression patterns of *TaAL* genes according to previous study [51]. The total RNA was isolated using RNApure Plant Kit (Vazyme), and the first-strand cDNA was synthesized from 1 µg of total RNA using Prescript III RT ProMix (CISTRO). The real-time PCR was performed using gene-specific primers (Table S10) with 2× Ultra SYBR Green qPCR Mix (CISTRO), and the *TaActin* gene was selected as a reference control. The real-time PCR cycling parameters were 95 ℃ for 30 s, followed by 45 cycles at 95 ℃ for 5 s and 60 ℃ for 30 s, with a melting curve analysis. All reactions were performed in three technical and biological replicates. The relative expression levels of target genes were calculated using the 2^−△△CT^ method [52].

### Association analysis of *TaAL1-B* gene haplotypes with drought-related traits in wheat

The 681 wheat resequencing data in Wheat Union database [22] (http://wheat.cau.edu.cn/WheatUnion/) was used to analyze the haplotypes of *TaAL1-B* gene. PlantRegMap database [53] (https://plantregmap.gao-lab.org/index.php) was used to identify transcription factor binding site in the 1500 bp promoter sequences of *TaAL1-B*. The phenotypic data of 39 wheat survival rate under drought stress were obtained from previous study [23].

### Electronic supplementary material

Below is the link to the electronic supplementary material.

Supplementary Material 1

Supplementary Material 2

Supplementary Material 3

Supplementary Material 4

Supplementary Material 5

Supplementary Material 6

## Data Availability

No datasets were generated or analysed during the current study.

## Abbreviations

AL: Alfin-like
CDS: Coding sequence
HMM: Hidden Markov Model
MBS: MYB binding site
ABRE: ABA-responsive element
CGTCA-Motif: MeJA-responsive element
