# Supplementary figures and images for: Comprehensive analysis of Alfin-like transcription factors associated with drought and salt stresses in wheat (Triticum aestivum L.)
Source: BMC Genomics. 2024 Jul 17;25:701. doi: 10.1186/s12864-024-10557-y (PMC11256656; doi:10.1186/s12864-024-10557-y)

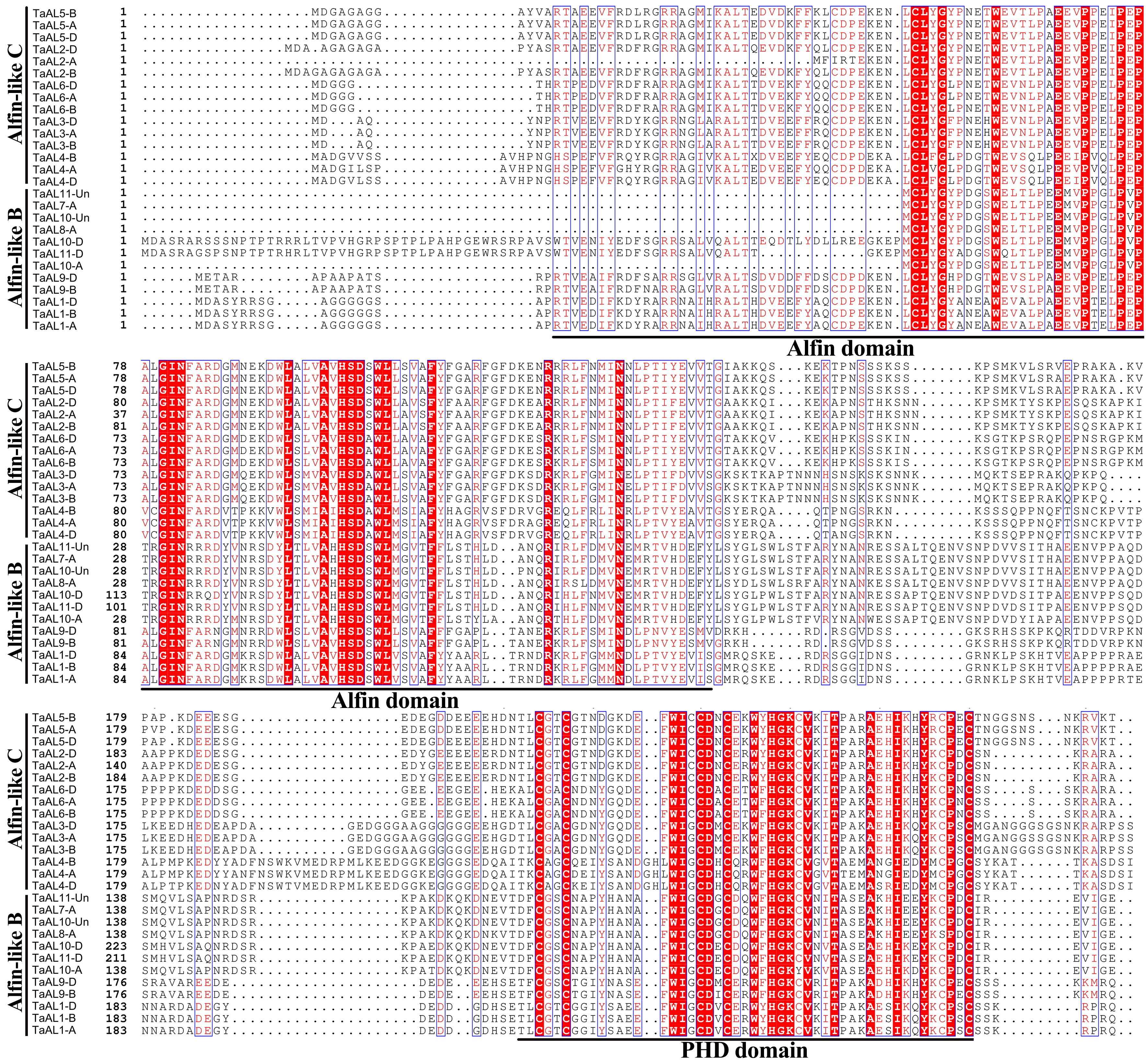

Supplement: Supplementary file 1 — Supplementary Material 1 [file 12864_2024_10557_MOESM1_ESM.jpg]

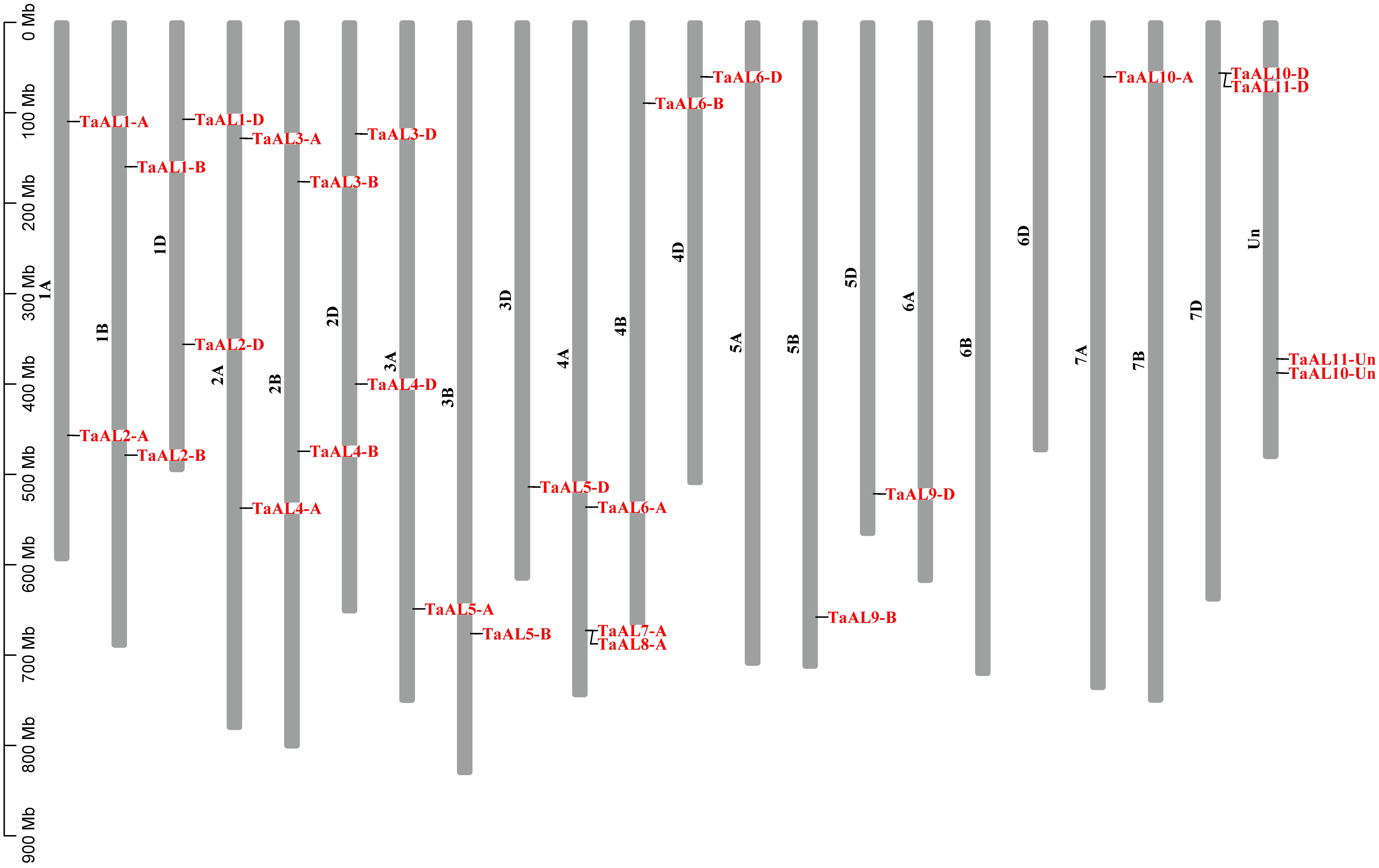

Supplement: Supplementary file 2 — Supplementary Material 2 [file 12864_2024_10557_MOESM2_ESM.jpg]

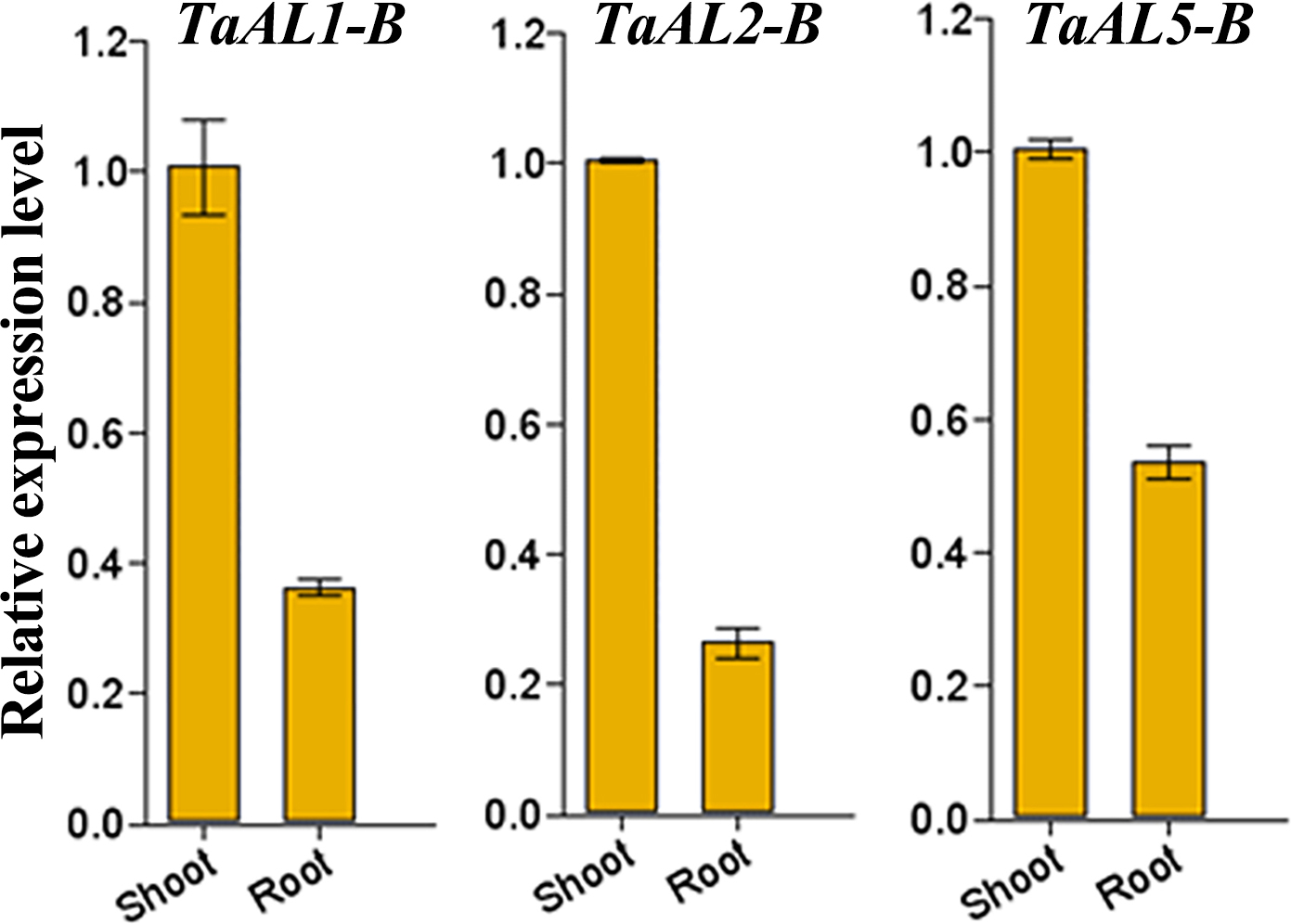

Supplement: Supplementary file 3 — Supplementary Material 3 [file 12864_2024_10557_MOESM3_ESM.jpg]

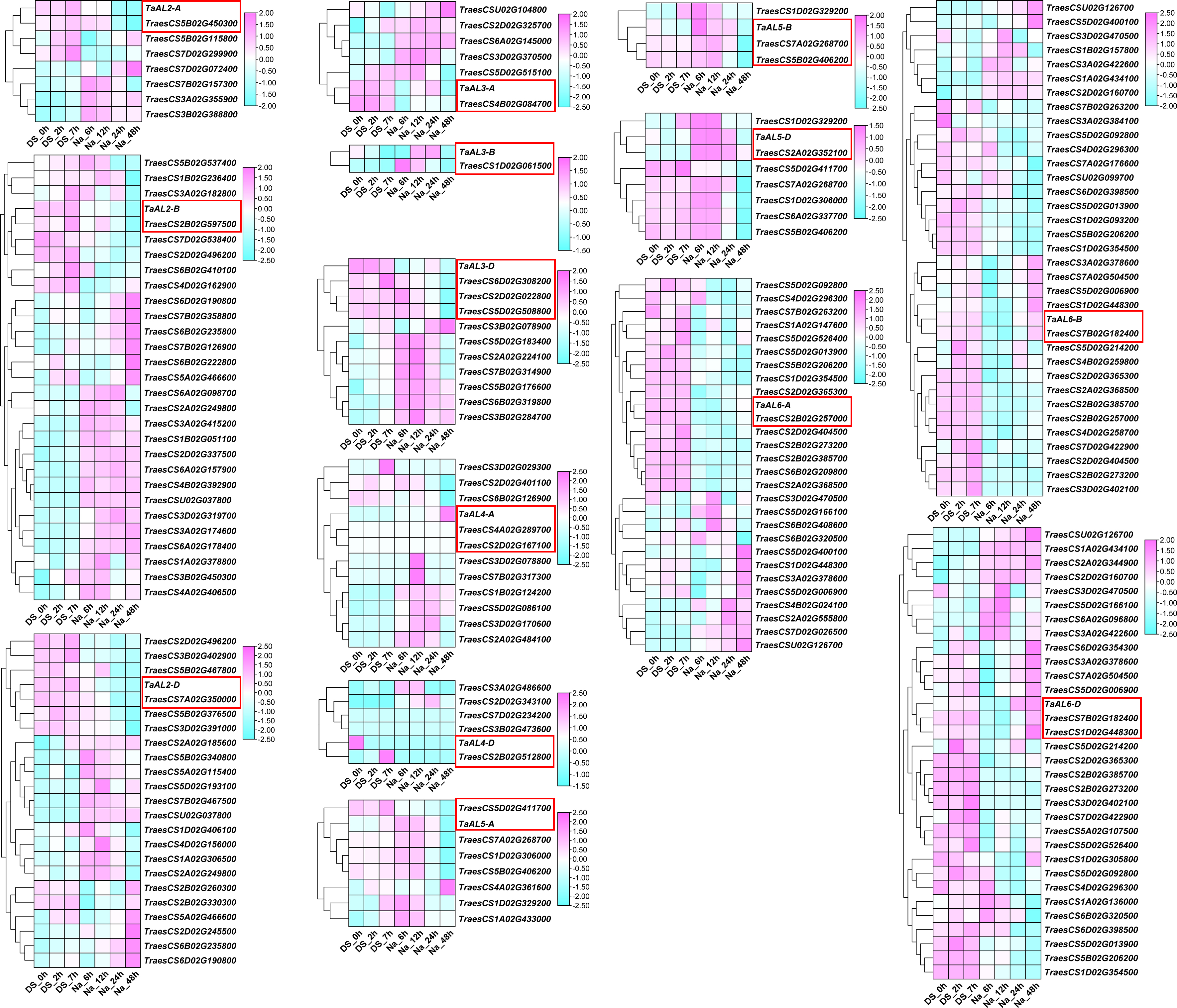

Supplement: Supplementary file 4 — Supplementary Material 4 [file 12864_2024_10557_MOESM4_ESM.jpg]
