# Supplementary material for: Comprehensive analysis of Alfin-like transcription factors associated with drought and salt stresses in wheat (Triticum aestivum L.)
Source: BMC Genomics. 2024 Jul 17;25:701. doi: 10.1186/s12864-024-10557-y (PMC11256656; doi:10.1186/s12864-024-10557-y)
Supplement: Supplementary file 5 — Supplementary Material 5 [file 12864_2024_10557_MOESM5_ESM.docx]

**Supplementary material**

Table S1. The characteristics of *Alfin-like* genes in wheat.

Table S2. Paralogous *TaAL* gene pairs in wheat.

Table S3. The log2 (FPKM) values of *TaAL* genes under drought and salt stresses.

Table S4. The interacting protein of TaALs.

Table S5. The downstream target genes of TaALs.

Table S6. The sequence and position of AL bingding site on the promoter of TaAL's downstream target genes.

Table S7. Variation sites and genotypes of *TaAL1-B* gene in 681 wheat materials.

Table S8. The information of the haplotype type and their genotypes distribution of *TaAL1-B* alleles.

Table S9. Wheat accessions and their drought tolerance phenotype (Survival rate, SR) .

Table S10. Specific primers used in this study.

Fig. S1. Multiple sequence alignment of the conserved domains of *TaAL* genes in wheat.

Fig. S2. Chromosomal locations of *TaAL* genes in wheat.

Fig. S3. Real-time PCR analysis of the expression profiles of *TaAL* genes in shoot and root tissues during wheat seedling stage.

Fig. S4. Expression patterns of downstream target gene of TaAL proteins. Fragments per kilobase of exon per million mapped fragments (FPKM) values were used to measure the expression levels of genes.
